# Effects of Sub-Inhibitory Rifampicin, Minocycline, and Dalbavancin on Early Biofilm Formation and Transcriptional Responses in *Staphylococcus aureus* SA113

**DOI:** 10.3390/microorganisms14092101

**Published:** 2026-09-19

**Authors:** Adam Bieda, Sabine Illner, Volkmar Senz, Stefan Oschatz, Niels Grabow, Micha Löbermann, Emil Christian Reisinger, Martina Sombetzki

**Affiliations:** 1 Department of Internal Medicine, Division of Infectious Diseases, Nephrology, Endocrinology and Tropical Medicine, University Medical Center Rostock, 18057 Rostock, Germany; adam.bieda@med.uni-rostock.de (A.B.); micha.loebermann@med.uni-rostock.de (M.L.); emil.reisinger@med.uni-rostock.de (E.C.R.); 2 Institute for Biomedical Engineering, University Medical Center Rostock, 18109 Rostock, Germany; sabine.illner@uni-rostock.de (S.I.); volkmar.senz@uni-rostock.de (V.S.); stefan.oschatz@uni-rostock.de (S.O.); niels.grabow@uni-rostock.de (N.G.)

**Keywords:** implant-associated infection, sub-inhibitory antibiotic exposure, *Staphylococcus aureus*, biofilm regulation, dalbavancin anti-biofilm activity, abiotic surfaces

## Abstract

Implant-associated infections are often caused by biofilm-forming pathogens such as *Staphylococcus (S.) aureus*. While local antibiotic delivery systems aim to prevent adhesion and biofilm establishment, declining drug concentrations may result in sub-inhibitory exposure, which can modulate bacterial adaptation linked to antibiotic resistance, biofilm formation, and regulatory responses. We investigated the effects of sub-inhibitory antibiotic exposure on biofilm formation in *S. aureus* SA113 and associated transcriptional responses. The biofilm-producing strain *S. aureus* SA113 was exposed to sub-inhibitory concentrations of rifampicin, minocycline, and dalbavancin during early biofilm formation. Phenotypic effects were assessed by crystal violet staining, enumeration of colony-forming units, and scanning electron microscopy, while transcriptional responses were analyzed by qPCR. Despite stable counts of culturable adherent bacteria, sub-inhibitory antibiotic exposure differentially altered biofilm formation and transcriptional responses. Rifampicin was associated with increased biomass at higher sub-inhibitory concentrations and increased early expression of *icaA*, *icaD* (+4.2 log_2_) and *fnbA* (+2.7 log_2_) at 1/2× MIC. Minocycline reduced biomass at lower concentrations with partial recovery toward control levels at 1/2× MIC, while transcriptional analysis at 1/4× MIC after 6 h showed increased expression of *icaA*, *icaD* (+2.1 log_2_) and *fnbA* (+4.3 log_2_). Dalbavancin induced a distinct transcriptional response characterized by increased expression of *vraS* (+1.1 log_2_) and *lrgA* (+1.8 log_2_), without induction of matrix-associated genes, while adherent biofilm biomass was reduced to 42.9% at 1/2× MIC. Morphologically, this was associated with compact aggregates rather than diffuse biofilm structures. Sub-inhibitory antibiotic exposure differentially modulated early biofilm formation in SA113 in a drug-specific manner. Overall, the distinct dalbavancin-associated response may be relevant for the development of preventive local drug-delivery systems.

## 1. Introduction

Implant-associated infections represent a major clinical challenge, predominantly driven by biofilm-forming pathogens, including *staphylococci, streptococci, enterococci,* and Gram-negative bacteria such as *Pseudomonas aeruginosa*. *Staphylococcus* (*S.*) *aureus* is a leading cause of device-associated infections, accounting for approximately 20–40% of cardiac implantable electronic device infections and prosthetic joint infections, while also substantially contributing to catheter-related bloodstream infections [1,2,3]. Following attachment to abiotic surfaces, *S. aureus* can establish biofilms characterized by an extracellular matrix, metabolic heterogeneity and increased tolerance to antimicrobial agents [4,5,6], frequently complicating eradication and necessitating surgical intervention [7].

Biofilm formation is a highly regulated, multistage process involving initial surface attachment, mediated by cell-wall-anchored adhesins, including fibronectin-binding proteins (FnBPA/B), and by autolytic processes contributing to extracellular DNA release [8,9]. Intercellular accumulation can involve the polysaccharide intercellular adhesin (PIA/PNAG), synthesized by the *icaADBC* operon, which is regulated by the transcriptional repressor IcaR [10,11]. Overall, these processes are controlled by regulatory systems, such as staphylococcal accessory regulator A (SarA), alternative sigma factor B (σB), quorum-sensing systems including accessory gene regulator (Agr), and stress-responsive pathways such as vancomycin resistance-associated regulatory system (VraSR), which integrate environmental and antibiotic stress signals to coordinate adhesion, matrix production, and biofilm stability [12,13,14]. During biofilm maturation, structural heterogeneity and altered bacterial physiology further contribute to antimicrobial tolerance and persistence [15,16,17].

Strong biofilm-producing phenotypes are frequently observed among clinical *S. aureus* isolates, including isolates associated with device- and implant-related infections [18,19,20,21,22]. *S. aureus* SA113 is a well-established, strongly biofilm-forming model strain for studying staphylococcal biofilm formation [10,23,24]. Its dysfunctional Agr system is associated with reduced biofilm dispersal and pronounced surface-associated accumulation [12]. As several studies have demonstrated, Agr dysfunction has also been associated with persistent and unfavorable outcomes in invasive *S. aureus* infections [25,26].

Because biofilm-associated infections frequently require prolonged antibiotic therapy and surgical intervention, including implant removal [4,18], preventive strategies targeting the early stages of bacterial adhesion and subsequent biofilm formation are of high translational relevance for the development of preventive biomaterial coatings [18,27]. Local antibiotic delivery from biomaterial surfaces represents one such strategy. Bioresorbable polymer-based systems can provide local antibiotic release at the implant interface [27,28]. The rifampicin- and minocycline-containing TYRX antibacterial envelope illustrates the clinical application of this concept [29].

However, declining local drug concentrations may eventually result in insufficient antimicrobial exposure [27]. Increasing evidence indicates that sub-inhibitory concentrations not only fail to inhibit bacterial growth but can actively modulate bacterial behavior, including biofilm formation, virulence factor expression, and the emergence of antibiotic resistance [30,31]. These responses appear to depend on both antibiotic class and strain-specific regulatory characteristics. While highly effective at inhibitory concentrations, sub-inhibitory levels of rifampicin and tetracyclines have been reported to promote biofilm formation in a strain- and concentration-dependent manner, including in weak or moderate biofilm producers [32,33]. Similar effects have been described for β-lactams [34,35], clindamycin [36] and several other antibiotics.

Dalbavancin is a long-acting lipoglycopeptide antibiotic [37] active against Gram-positive bacteria, including methicillin-resistant *S. aureus* (MRSA) and methicillin-resistant *S. epidermidis* (MRSE) [38,39], which inhibits cell-wall synthesis by binding to D-Ala-D-Ala termini of peptidoglycan precursors [40]. Dalbavancin has demonstrated activity against established staphylococcal biofilms [38,41,42] and effective distribution into deep tissues [37]. In addition to its established use in skin and soft tissue infections, it is increasingly investigated in bone and implant-associated infections and exhibits superior activity compared to vancomycin in several models [41,42,43]. A previous in vivo study further demonstrated that local dalbavancin release significantly reduced bacterial burden and associated inflammatory responses following implantation [44]. However, the regulatory responses to sub-inhibitory dalbavancin exposure during early adhesion and biofilm formation remain poorly characterized. More broadly, antibiotic-specific phenotypic and transcriptional responses during early surface colonization under sustained sub-inhibitory exposure remain insufficiently understood.

In this study, we investigated how exposure to sub-inhibitory concentrations of rifampicin, minocycline, and dalbavancin affects biofilm formation and associated transcriptional responses in the *agr*-deficient, strongly biofilm-forming strain *S. aureus* SA113. Using quantitative, structural, and transcriptional analyses, we assessed antibiotic-specific effects on adherent biofilm biomass, culturable adherent bacterial counts, surface colonization, and biofilm-associated gene expression. We hypothesized that differences in antibiotic mode of action would be associated with distinct phenotypic and transcriptional response patterns during early biofilm development, with particular emphasis on dalbavancin-associated cell-wall stress responses.

## 2. Materials and Methods

### 2.1. Bacterial Cultivation

*Staphylococcus aureus* SA113 (ATCC 35556) was purchased from the American Type Culture Collection (ATCC, Manassas, VA, USA) and stored at −80 °C in tryptic soy broth (TSB) (Sigma-Aldrich, Darmstadt, Germany), supplemented with 15% glycerol. Bacterial material from glycerol stocks was streaked onto Columbia sheep blood agar (5%) (Becton Dickinson, Franklin Lakes, NJ, USA). Single colonies were inoculated into TSB and incubated overnight at 37 °C in 5% CO_2_.

### 2.2. Antibiotic Preparation

Antibiotics were purchased from Sigma-Aldrich, Germany. Stock solutions were prepared at 10.24 mg/mL in DMSO and stored at −20 °C. Working solutions were prepared by diluting the stocks in sterile water and stored at 4 °C. The residual DMSO concentration in the final assay was ≤0.05% (*v*/*v*). Prior to use, antibiotic solutions were briefly pulse-centrifuged to collect condensed liquid and subsequently vortexed thoroughly to ensure homogeneous mixing. For Dalbavancin-containing solutions, polysorbate-80 was added at a final concentration of 0.002%, according to recent EUCAST recommendations [45].

### 2.3. Broth Microdilution

Determination of the minimum inhibitory concentration (MIC) was carried out in flat-bottom polystyrene 96-well microtiter plates (Greiner Bio-One, Kremsmünster, Austria) according to the broth microdilution method. Bacterial overnight cultures were diluted 1:20 in fresh TSB and grown to an optical density (OD600) of 0.2. Bacterial suspensions were then diluted 1:300 in cation-adjusted Mueller–Hinton broth (CAMHB) (Sigma-Aldrich, Germany) to obtain a concentration of 5 × 10^5^ CFU/mL. For biofilm experiments, MIC determination was carried out using TSB under the same experimental conditions. Briefly, 10 µL of a 20× antibiotic solution was mixed with 10 µL of the corresponding diluent (sterile water or 0.04% polysorbate-80), yielding a 10× antibiotic solution containing 0.02% polysorbate-80 where applicable. Two-fold serial dilutions were then prepared by transferring 10 µL into 10 µL of fresh diluent (sterile water or 0.02% polysorbate-80). After removal of 10 µL from the final well to equalize volumes, 90 µL of bacterial suspension (5 × 10^5^ CFU/mL) was added to each well, yielding the intended final antibiotic concentrations and 0.002% polysorbate-80 where applicable. Bacteria were then incubated for 24 h at 37 °C and 5% CO_2_. Bacteria receiving the corresponding antibiotic-free diluent served as growth controls. Sterile medium supplemented with the terminal dilution of the respective antibiotic served as a sterility control. MIC was defined as the lowest antibiotic concentration at which no visible bacterial growth was observed.

### 2.4. Biofilm Model

To assess the effects of inhibitory and sub-inhibitory antibiotic exposure on early biofilm formation, bacteria were inoculated into TSB containing the respective antibiotics or reference controls at the start of incubation (see Section 2.3). Antibiotic concentrations were prepared as described above by performing two-fold serial dilutions directly in flat-bottom 96-well polystyrene microtiter plates (Greiner Bio-One, Austria). Assays with dalbavancin were supplemented with polysorbate-80 at a final concentration of 0.002%. Biofilm assays were performed using an inoculum of 5 × 10^5^ CFU/mL. Unless otherwise specified, bacteria were incubated in the presence of the respective antibiotics for 24 h at 37 °C and 5% CO_2_ prior to downstream analyses of the resulting biofilms. Relative antibiotic concentrations (referred to as 2× to 1/32× MIC) used in biofilm experiments were defined based on the assay-specific MIC values determined in TSB under the corresponding conditions described above.

### 2.5. Crystal Violet Biofilm Quantification

Crystal violet staining was performed as adapted from Kwasny and Opperman [46]. After 24 h of incubation, the supernatants were discarded and loosely adherent bacteria were removed by washing the wells three times with 100 µL of PBS using gentle pipetting. Subsequently, biofilms were fixed by drying at 60 °C for 60 min and stained with 50 µL of a 0.06% crystal violet solution. After 5 min, the excess stain was aspirated and removed by washing three times using sterile distilled water. Crystal violet (CV) was eluted using 100 µL of 30% acetic acid. The supernatants were transferred to a new plate and serially diluted if required using 30% acetic acid. Absorbance was measured via spectrophotometry (FLUOstar Omega, BMG LABTECH, Ortenberg, Germany) at 590 nm and corrected for the dilution factor. CV staining was repeated in five biological replicates. Untreated bacteria receiving sterile water or 0.002% polysorbate-80 served as the respective reference controls.

### 2.6. Determination of Colony-Forming Units

Culturable adherent bacterial cell counts were determined by colony-forming unit (CFU) enumeration. After 24 h of incubation under the conditions described in Section 2.4, the supernatants were discarded and loosely adherent bacteria were removed by gently washing the wells three times with 100 µL of PBS. Then, biofilms were resuspended in 100 µL PBS by thorough mixing and scratching of the well bottom using a pipette tip. For MIC conditions (1×–2× MIC), 50 µL was plated directly, whereas the remaining 50 µL was first diluted 1:10 in PBS, sonicated for 5 min at 45 kHz (VWR USC 200T, VWR International, Leuven, Belgium), and subsequently serially diluted prior to plating. For sub-inhibitory conditions (<1× MIC), the entire 100 µL was diluted 1:10, sonicated for 5 min, then serially diluted using PBS and plated on tryptic soy agar. After 24 h to 48 h of incubation at 37 °C and 5% CO_2_, bacterial colonies were counted and CFU/mL of the recovered biofilm suspension was calculated from the colony count, the respective dilution factor, and the plated volume.

### 2.7. Scanning Electron Microscopy

For scanning electron microscopy (SEM), bacteria were cultivated on 6 mm Thermanox coverslips (Thermo Fisher Scientific, Waltham, MA, USA), under the conditions described in Section 2.4. Excess culture medium was removed, and biofilms were gently washed three times with PBS, unless stated otherwise. Samples were fixed overnight at 4 °C in a primary fixative consisting of 2% glutaraldehyde, 2% paraformaldehyde, and 0.15% alcian blue in 0.1 M sodium cacodylate buffer (pH 7.2–7.4). After three gentle washes with sodium cacodylate buffer, specimens were post-fixed for 1.5 h in 1% osmium tetroxide and 1% tannic acid, as adapted from Wells et al. [47]. After thorough washing with deionized water, the samples were dehydrated in a graded ethanol series (50%, 70%, 90%, 95%, 100%, 100%) for 15 min per step. Subsequently, specimens were incubated for 5 min in a 1:1 mixture of absolute ethanol and hexamethyldisilazane (HMDS), followed by immersion in 100% HMDS and overnight air-drying. Mounted specimens were coated with gold using a sputter coater (Agar Scientific, Rotherham, UK). Scanning electron microscopy was performed using a Quanta FEG 250 (FEI Company, Hillsboro, OH, USA), operated at 10 kV under high vacuum conditions using an ETD detector, with a working distance of approximately 10 mm.

### 2.8. RNA Isolation and cDNA Synthesis

Bacteria were incubated under the biofilm model conditions described in Section 2.4 in the presence of the respective antibiotics. After 6 h and 24 h of incubation, biofilm-associated bacterial material was collected by pooling eight wells per condition to obtain sufficient biomass for subsequent RNA extraction. Transcriptional analyses were generally performed at 1/2× MIC; for minocycline at 6 h, the 1/2× MIC condition did not yield evaluable qPCR data; therefore, the next lower concentration (1/4× MIC) was analyzed. After removal of the culture medium, wells were briefly rinsed with a small volume of Tris-HCl buffer. Biofilm-associated material was resuspended in ice-cold 200 mM Tris-HCl and centrifuged for 5 min at 10,000–12,000× *g*. The supernatant was aspirated, and samples were snap-frozen and stored at −80 °C until further processing. Bacterial lysis was performed enzymatically using lysostaphin (Sigma-Aldrich, Germany) (100 µg/mL, in 200 mM Tris-HCl, pH 8) for 15 min at 37 °C. Immediately afterward, the chaotropic RNA Lysis Buffer from the Quick-RNA Fungal/Bacterial Miniprep Kit (Zymo Research, California, USA) was added to stabilize the released RNA and inactivate RNases. Samples were then vigorously vortexed, and three freeze–thaw cycles alternating between liquid nitrogen and a 60 °C water bath were applied, as adapted from Beltrame et al. [48].

Total RNA was then isolated and purified using the Quick-RNA Fungal/Bacterial Miniprep Kit (Zymo Research, USA). RNA was eluted in 25 µL of RNase-free water. RNA purity was assessed by spectrophotometric measurement at 260/280 nm (Colibri, Berthold Technologies, Bad Wildbad, Germany), and RNA integrity was checked by agarose gel electrophoresis. Genomic DNA was removed using the TURBO DNase Kit (Thermo Fisher Scientific, USA) according to the manufacturer’s instructions. RNA concentration was quantified using the Qubit RNA Broad Range Quantification Kit (Thermo Fisher Scientific, USA), and a total of 100 ng of RNA was reverse transcribed using the High-Capacity cDNA Reverse Transcription Kit (Thermo Fisher Scientific, USA).

### 2.9. qPCR and Gene Expression Analysis

Gene expression levels were determined by quantitative polymerase chain reaction (qPCR) using cDNA corresponding to 2.5 ng of reverse-transcribed RNA input per reaction in a total volume of 10 μL using PowerTrack SYBR Green Master Mix (Thermo Fisher Scientific, USA) according to the manufacturer’s instructions.

Target genes were selected to represent key pathways involved in biofilm formation and regulation, including PIA/PNAG synthesis and regulation (*icaA*, *icaD*, *icaR*), adhesion (*fnbA*), autolysis/remodeling (*lrgA*, *cidA*, *atlA*), and stress/global regulation (*vraS*, *sarA*, *sigB*). Primers were designed using NCBI Primer-BLAST based on the *S. aureus* NCTC 8325 genome (Accession: NZ_LS483365.1), the parental strain of SA113, and synthesized by Eurofins Genomics, Germany. Primer sequences and amplicon lengths are provided in Table 1. qPCR amplification was performed using QuantStudio 3 (Thermo Fisher Scientific, USA) under the following cycling conditions: initial denaturation at 95 °C for 2 min, followed by 40 cycles of 95 °C for 15 s and 60 °C for 1 min. Subsequently, melt curve analysis was performed to verify amplification specificity.

### 2.10. Statistical Analysis

Statistical analyses were performed using GraphPad Prism 10.6 (GraphPad Software, Boston, MA, USA) and Python 3.12. Normalized biofilm biomass values were compared against a theoretical control value of 100%, and two-tailed one-sample t-tests were performed with Holm−Bonferroni correction applied within each antibiotic concentration series. Gene expression differences for the 6-h transcriptional data were analyzed on −ΔCt values using gene-wise linear models with treatment as a fixed effect and biological replicate as a blocking factor. Planned treatment-versus-control comparisons were performed, and *p*-values were adjusted using the Holm–Bonferroni correction within each gene. A *p*-value < 0.05 was considered statistically significant.

## 3. Results

### 3.1. Antibacterial Susceptibility Profile

General antibacterial susceptibility was determined under standard CAMHB conditions. MICs ranged from 0.06–0.125 μg/mL for rifampicin (RIF), 0.03–0.06 μg/mL for minocycline (MIN), 0.03–0.06 μg/mL for the rifampicin/minocycline combination (RIF/MIN), and 0.125–0.25 μg/mL for dalbavancin (DAL). In agreement with previous reports [49], the addition of 0.002% polysorbate-80 markedly lowered the measured dalbavancin MIC, consistent with reduced surface adsorption during susceptibility testing. According to EUCAST clinical breakpoints [45], the strain was classified as susceptible to all investigated antibiotics and showed no evidence of broader clinically relevant resistance.

As subsequent biofilm experiments were performed in TSB, inhibitory and sub-inhibitory exposure levels were defined relative to the corresponding MIC in TSB. Under these conditions, MICs were modestly elevated to 0.125–0.25 μg/mL for rifampicin, 0.06–0.125 μg/mL for minocycline, 0.06–0.125 μg/mL for the rifampicin/minocycline combination, and 0.5–1.0 μg/mL for dalbavancin. Biofilm experiments covered a dilution range spanning approximately 2× to 1/32× MIC.

### 3.2. Low-Level Polysorbate-80 Exposure Does Not Interfere with Overall Biofilm Formation

**Figure A1:**
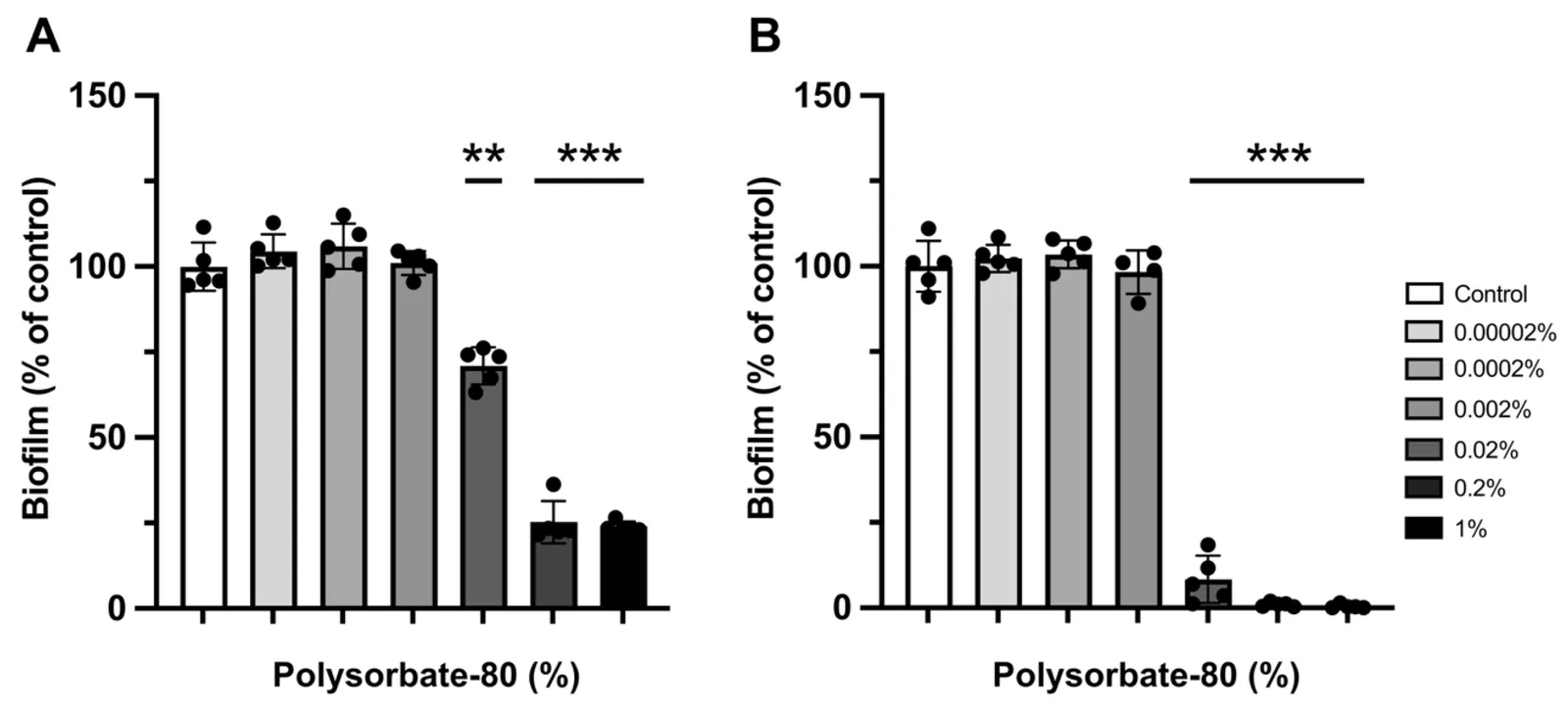
Low-level polysorbate-80 exposure does not affect crystal violet-based biofilm biomass quantification in *Staphylococcus aureus* SA113. Bacteria were cultivated for 24 h at 37 °C and 5% CO2 in tryptic soy broth supplemented with increasing concentrations of polysorbate-80. Total stainable biomass without washing (**A**) and adherent biofilm biomass after three PBS washes prior to staining (**B**) were quantified by crystal violet staining and are presented relative to the polysorbate-80-free control condition (mock-treated with sterile water), defined as 100%. Data points represent five independent biological replicates, with bars indicating mean ± SD. Statistical analyses were performed using two-tailed one-sample t-tests with Holm–Bonferroni correction. Significance: *p* < 0.01 (**), *p* < 0.001 (***).

*S. aureus* SA113 displayed a pronounced capacity for biofilm formation, reaching total biomass values of up to OD_590_ ≈ 6.6 as quantified by crystal violet (CV) staining. For experimental validation, bacteria were incubated in serial dilutions of polysorbate-80 ranging from 1% to 0.00002%, corresponding to ±2 log_10_ of the concentration recommended for susceptibility testing with dalbavancin. While CV staining showed a pronounced reduction in total biofilm (not washed) and adherent biofilm (washed three times) biomass at polysorbate-80 concentrations ≥ 0.02%, no significant differences in biofilm biomass were observed compared to the untreated control at 0.002% and lower (Figure A1). These findings support the use of 0.002% polysorbate-80 as a suitable supplementation for comparative biofilm experiments in this strain.

### 3.3. Antibiotic Exposure Alters Adherent Biofilm Mass Without Major Differences in Culturable Bacterial Counts

**Figure 1:**
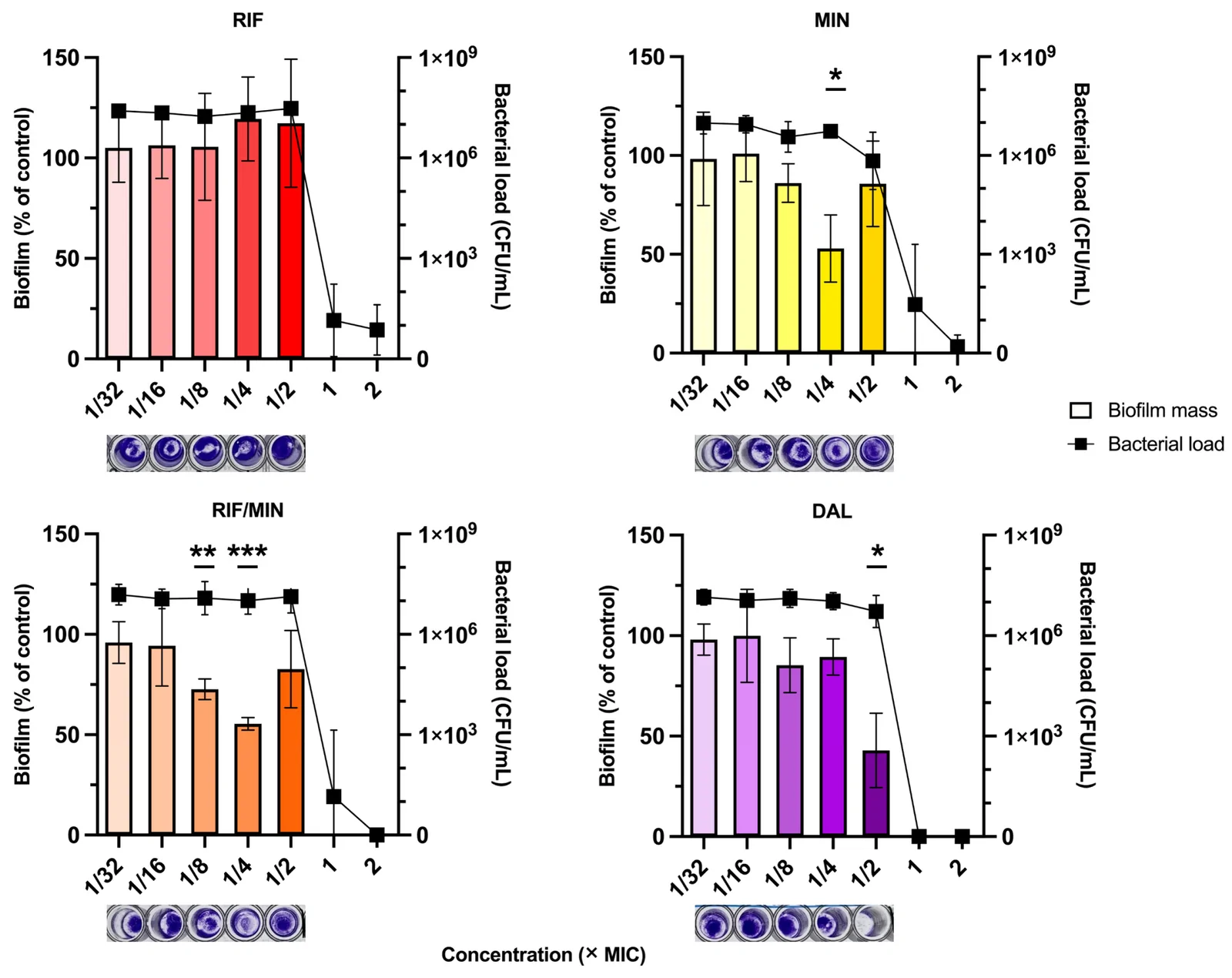
Sub-inhibitory antibiotic exposure differentially modulates adherent biofilm biomass in *Staphylococcus aureus* SA113. Bacteria were cultivated for 24 h at 37 °C and 5% CO_2_ in two-fold serial dilutions of rifampicin (RIF), minocycline (MIN), rifampicin/minocycline (RIF/MIN) (1:1), and dalbavancin (DAL). Adherent biofilm biomass after three PBS washes prior to staining was quantified by crystal violet staining and is presented relative to the respective antibiotic-free reference control condition, defined as 100% (sterile water for RIF-, MIN-, and RIF/MIN-exposed conditions; polysorbate-80 for DAL). Representative CV-stained wells are shown below each graph. Statistical analyses of CV data were performed using two-tailed one-sample t-tests with Holm–Bonferroni correction. Significance: *p* < 0.05 (*), *p* < 0.01 (**), *p* < 0.001 (***). CV data (bar plots) represent mean ± SD of five biological replicates. Culturable adherent bacteria were quantified by colony-forming unit (CFU) enumeration and are shown as black squares on the right *y*-axis, expressed as CFU/mL. CFU data represent mean ± SD of three biological replicates.

Crystal violet staining revealed distinct, concentration-dependent effects of the tested antibiotics on adherent biofilm biomass. At 1/32× and 1/16× MIC, no measurable changes in adherent biofilm biomass were observed for any of the tested antibiotics (Figure 1). At 1/4× and 1/2× MIC, rifampicin increased adherent biofilm mass, reaching 117.8% and 113.4% of the control level, respectively; however, these differences were not statistically significant. By contrast, minocycline exposure led to a biphasic response, reducing adherent biofilm biomass to 80.9% at 1/8× and 50.2% (*p* = 0.024) at 1/4× MIC, whereas biomass returned to 85.7% of the control level at 1/2× MIC, which may reflect a balance between impaired protein synthesis at lower concentrations and stress-induced biofilm adaptation at higher sub-inhibitory levels. Interestingly, although rifampicin/minocycline co-exposure significantly reduced biofilm biomass at 1/8× and 1/4× MIC compared with the untreated control, it did not consistently reduce biomass further than observed with minocycline mono-exposure. By contrast, dalbavancin did not significantly affect adherent biofilm biomass at 1/8× or 1/4× MIC, whereas a significant reduction in adherent biofilm mass was observed at 1/2× MIC, reaching 42.9% (*p* = 0.049) of the corresponding control. Taken together, antibiotic exposure induced drug- and concentration-dependent effects on adherent biofilm biomass, with a biphasic response observed for minocycline.

In parallel, culturable adherent bacterial cell counts were quantified by CFU enumeration (Figure 1). Across the investigated sub-inhibitory antibiotic conditions, CFU counts remained broadly comparable, ranging from 1–3 × 10^7^ CFU/mL, indicating that changes in adherent biofilm biomass were not accompanied by corresponding differences in culturable adherent bacterial counts.

### 3.4. Spatial Organization and Surface Colonization of Biofilms Differ by Treatment

**Figure 2:**
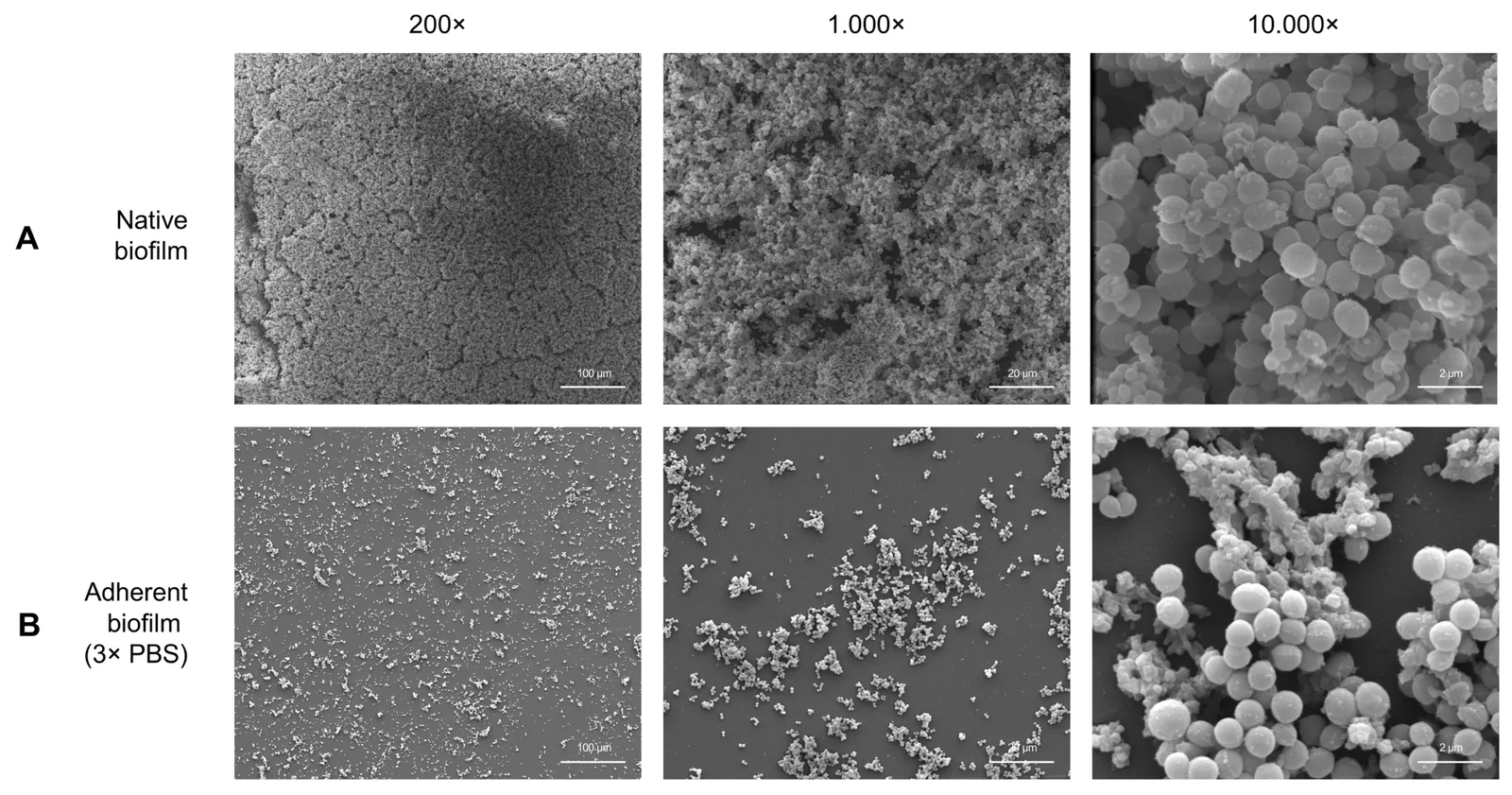
Scanning electron microscopy visualizes the structural organization of *Staphylococcus aureus* SA113 biofilms on Thermanox coverslips. Bacteria were cultivated for 24 h at 37 °C and 5% CO_2_ in tryptic soy broth on Thermanox coverslips. Representative images show the three-dimensional structure of unwashed biofilms (**A**) and the remaining adherent surface-associated bacterial material after three gentle PBS washes prior to fixation (**B**). Specimens were sputter-coated with gold and imaged at 10 kV using an ETD detector. Scale bars represent 100 µm, 20 µm, and 2 µm.

Scanning electron microscopy (SEM) was employed to complement crystal violet assays and to visualize surface colonization and gross biofilm morphology of *S. aureus* SA113 under sub-inhibitory antibiotic exposure. In unwashed samples, bacteria established uniform surface coverage with a three-dimensional architecture characterized by bacterial clusters, microchannel-like structures and structures consistent with extracellular material (Figure 2A). After three gentle PBS washes prior to fixation, surface colonization was markedly reduced, leaving predominantly single adherent cells and small clusters (Figure 2B). These washed samples therefore served as the morphological reference condition for assessing adherent surface-associated bacteria after antibiotic exposure.

**Figure 3:**
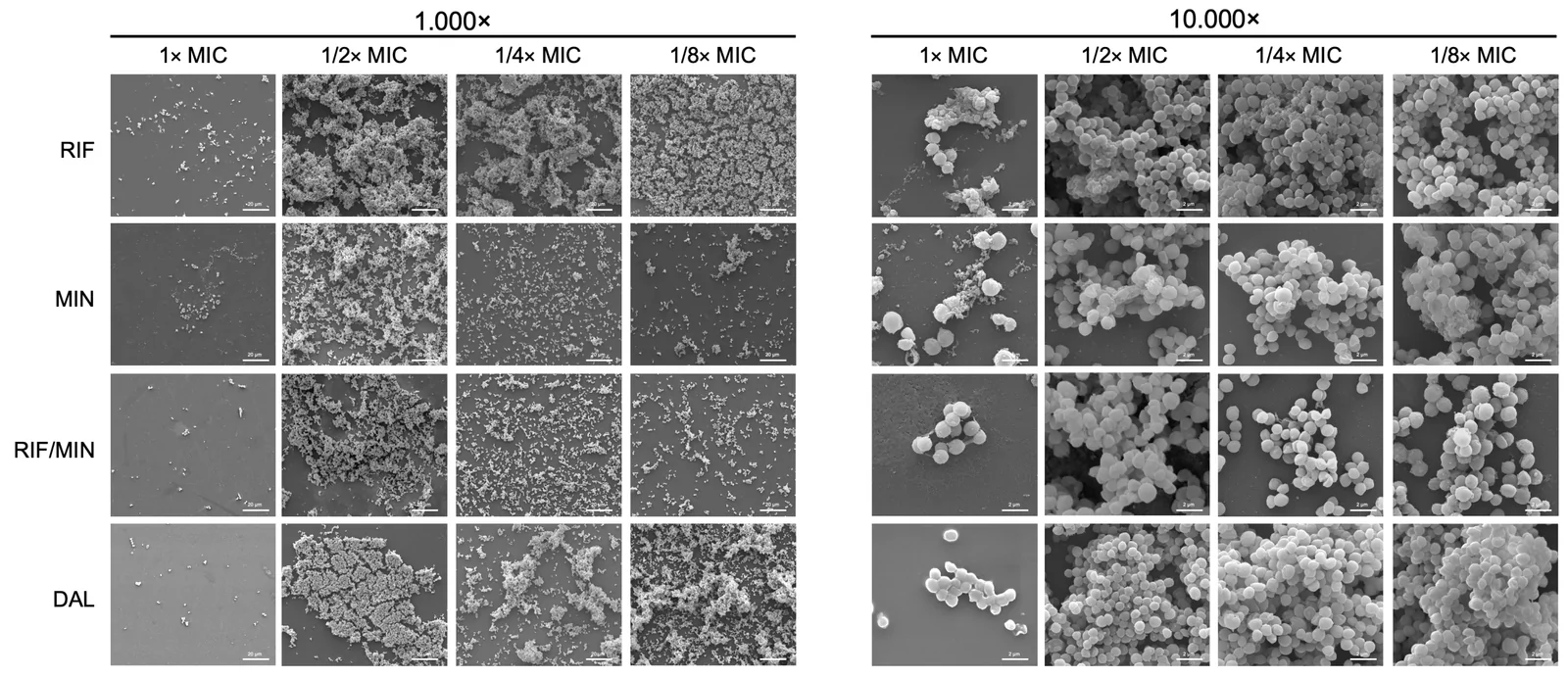
Sub-inhibitory antibiotic exposure reshapes surface colonization and biofilm morphology of *Staphylococcus aureus* SA113 on Thermanox coverslips. Bacteria were cultivated for 24 h at 37 °C and 5% CO_2_ in tryptic soy broth supplemented with rifampicin (RIF), minocycline (MIN), rifampicin/minocycline (RIF/MIN) (1:1), and dalbavancin (DAL) + 0.002% polysorbate-80 at 1×–1/8× MIC. Samples were washed with PBS prior to fixation. Representative images show antibiotic-specific and concentration-dependent differences in bacterial adherence and aggregation. Specimens were sputter-coated with gold and imaged at 10 kV using an ETD detector. Scale bars represent 20 µm and 2 µm.

Across the examined conditions, SEM did not reveal consistent gross morphological signs of severe structural damage, such as pronounced cell deformation or widespread disruption of bacterial cell shape. Occasional indentations or partially shrunken cells were observed, but these features were not consistently associated with a specific treatment and may reflect artifacts introduced during SEM preparation. Structures consistent with extracellular material were observed in some antibiotic-exposed samples, particularly around remaining bacterial aggregates, and appeared more prominent in rifampicin- and minocycline-exposed samples (Figure 3). However, SEM does not allow conclusions regarding cell-envelope integrity, bacterial viability and matrix composition. Accordingly, these structures may represent extracellular matrix components, cellular debris, or preparation-related artifacts.

### 3.5. Sub-Inhibitory Antibiotic Exposure Is Associated with Distinct Transcriptional Response Patterns During Early Biofilm Formation

**Figure 4:**
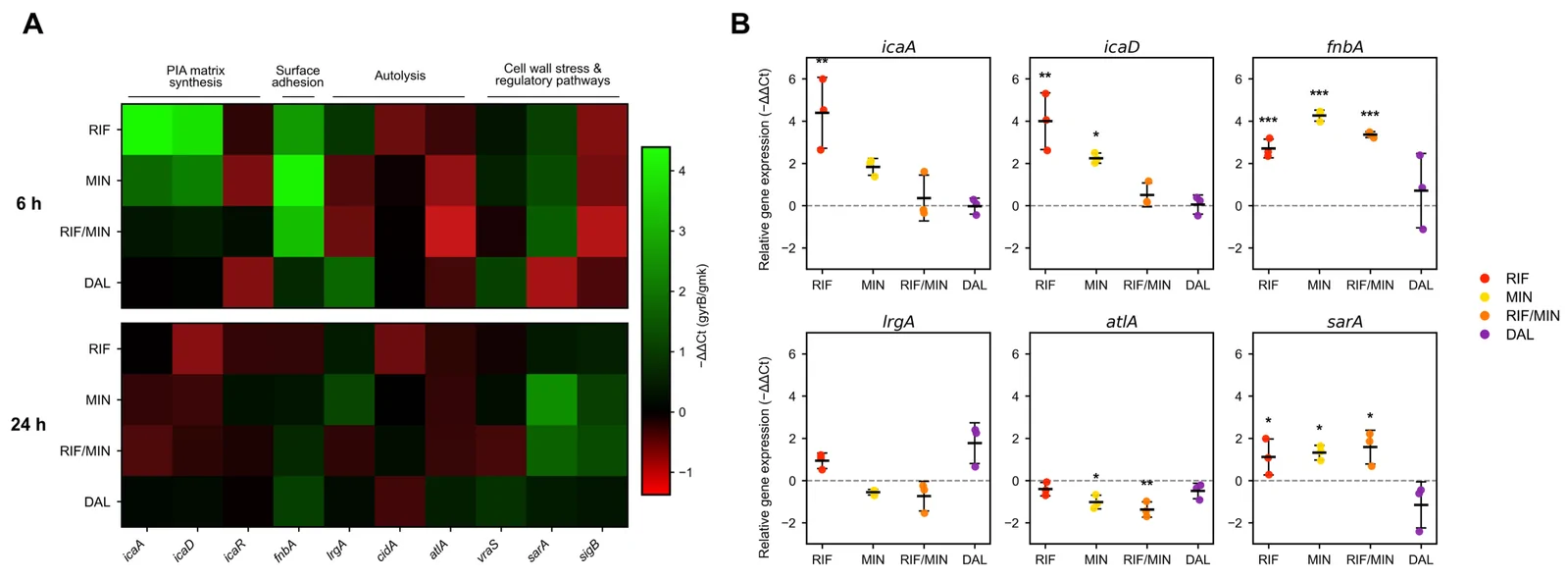
Antibiotic-specific transcriptional response patterns following sub-inhibitory exposure during early *Staphylococcus aureus* SA113 biofilm formation. (**A**) Heatmaps show mean log_2_ fold changes (−ΔΔCt) of selected biofilm- and stress-associated target genes following exposure to the respective sub-inhibitory concentrations of rifampicin (RIF), minocycline (MIN), rifampicin/minocycline (RIF/MIN; 1:1), or dalbavancin (DAL) at 6 h and 24 h. Green indicates upregulation and red indicates downregulation. Ct values were normalized to the reference genes *gyrB* and *gmk*, and −ΔΔCt were calculated relative to the respective time-matched vehicle control. RIF, MIN, and RIF/MIN were normalized to H_2_O controls; DAL was normalized to polysorbate-80 controls. Heatmap values represent mean −ΔΔCt from three biological replicates. For one 24-h H_2_O control replicate, Ct values were imputed from the mean of the two remaining H_2_O controls for descriptive −ΔΔCt analysis; no inferential statistics were performed at 24 h. (**B**) Gene-wise plots show individual biological replicates and mean ± SD for selected target genes at 6 h. Statistical analyses were performed on −ΔCt values using gene-wise linear models with biological replicate included as a blocking factor. Planned treatment-versus-control comparisons were adjusted using the Holm–Bonferroni method. Significance: *p* < 0.05 (*), *p* < 0.01 (**), *p* < 0.001 (***).

To investigate antibiotic-induced transcriptional alterations in biofilm formation, transcriptional analyses were performed at the highest evaluable sub-inhibitory concentration, capturing transcriptional responses during an active early biofilm-forming phase under high antibiotic stress. Selection of this concentration was not based on the magnitude of the phenotypic response observed in the CV assay. Relative gene expression was reported as log_2_ fold change (−ΔΔCt) and summarized as heatmaps illustrating compound-specific and time-dependent transcriptional profiles (Figure 4A). Selected gene-wise plots displaying individual replicates are presented in Figure 4B. Individual −ΔCt values for all target genes and biological replicates are provided in Supplementary Table S1.

At 6 h, rifampicin exposure was associated with pronounced changes in selected biofilm-associated genes, including moderate upregulation of *sarA* together with strong upregulation of the PIA/PNAG-associated genes *icaA* and *icaD* (+4.4 log_2_; +4.0 log_2_) and increased transcript abundance of the adhesion-associated gene *fnbA* (+2.7 log_2_) (Figure 4A,B). Minocycline showed a similar but partially distinct pattern, with moderately increased *sarA* transcript abundance, lower-magnitude upregulation of *icaA* and *icaD* (+1.8 log_2_; +2.3 log_2_), and more pronounced upregulation of *fnbA* (+4.3 log_2_). The concomitant increases in *sarA* and matrix- or adhesion-associated target genes occurred alongside antibiotic-dependent differences in adherent biofilm biomass and surface colonization observed in the crystal violet assays and SEM. In contrast, rifampicin/minocycline co-exposure showed limited induction of *icaA* and *icaD* (≤0.5 log_2_) at the sampled time point despite elevated *sarA* levels, whereas *fnbA* remained robustly upregulated (+3.4 log_2_).

Dalbavancin exposure was characterized by increased *vraS* (+1.1 log_2_) and *lrgA* (+1.8 log_2_) transcript abundance. In contrast, *sarA* expression was not increased, and induction of the selected matrix-associated genes remained absent (*icaA*: −0.02 log_2_; *icaD*: +0.05 log_2_), whereas *fnbA* showed only a slight mean increase in transcript abundance (+0.7 log_2_). Furthermore, neither *atlA* nor *cidA* showed increased transcript abundance. This transcriptional profile coincided with the distinct clustered surface colonization observed by SEM following dalbavancin exposure.

Compared with the pronounced transcriptional responses observed after 6 h, treatment-associated transcriptional differences were generally attenuated after 24 h (Figure 4A). While basal transcript abundance (−ΔCt) of *icaA* and *icaD* remained largely stable in the control groups, treatment-associated changes showed slight downregulation in rifampicin- and minocycline-treated bacteria at 24 h (Figure 4A). The strong early induction of *fnbA* was substantially reduced at 24 h, despite sustained *sarA* expression in rifampicin- and minocycline-treated bacteria (Figure 4A). The reduction in treatment-associated *fnbA* expression at 24 h coincided with increased basal transcript abundance (−ΔCt) in the control group. In contrast, the transcript abundance of the autolysis-associated genes *lrgA*, *cidA*, and *atlA*, as well as stress-associated genes *sarA* and *sigB,* was reduced at 24 h. Overall, several target genes showed attenuated treatment-associated transcriptional responses at 24 h (Figure 4).

**Figure A2:**
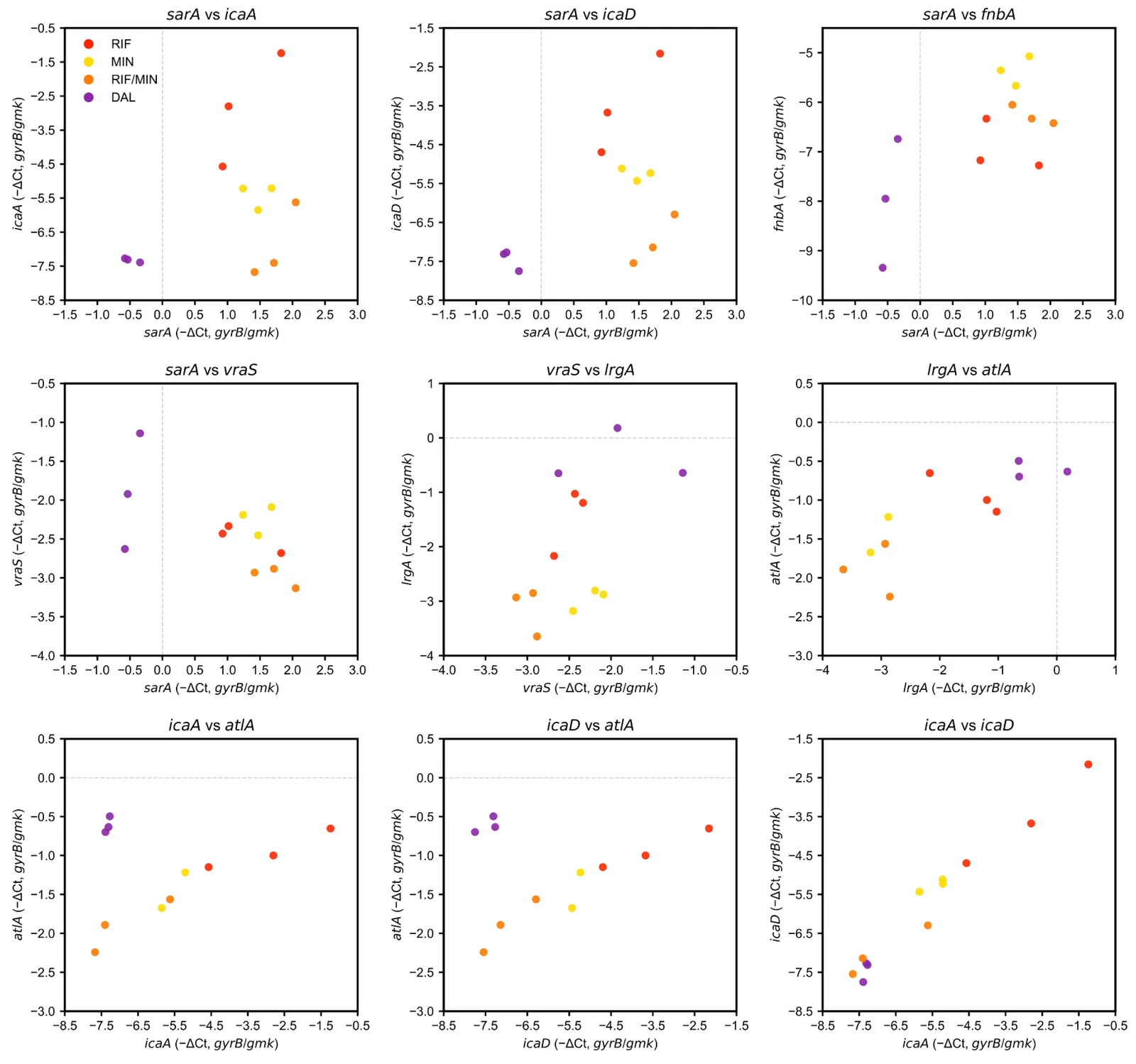
Targeted scatter plot analysis allows exploratory assessment of pairwise expression relationships among biofilm-associated genes. Scatter plots show pairwise relationships between selected biofilm-associated genes based on normalized qPCR expression values (−ΔCt) at 6 h following sub-inhibitory antibiotic exposure. Expression levels were normalized to the reference genes *gyrB* and *gmk*. Each data point represents one biological replicate treated with rifampicin (RIF), rifampicin/minocycline (RIF/MIN) and dalbavancin (DAL) at 1/2× MIC or minocycline (MIN) at 1/4× MIC (*n* = 3 per treatment). Selected gene pairs were chosen to explore relationships between adhesion-associated (*icaA*, *icaD*, *fnbA*), regulatory (*sarA*, *vraS*), and autolysis-associated (*lrgA*, *atlA*) pathways.

**Figure A3:**
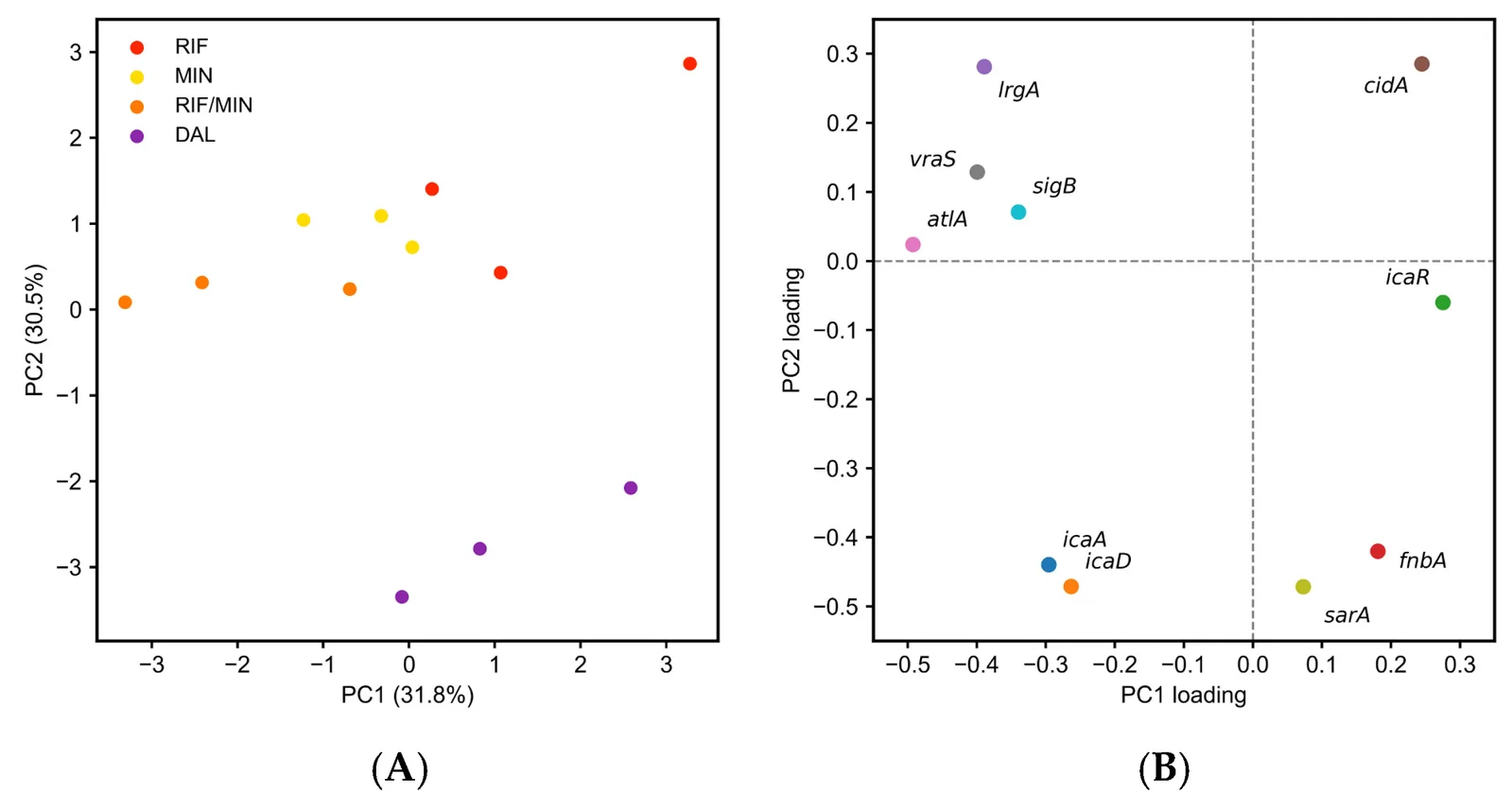
Exploratory PCA supports distinct transcriptional responses to sub-inhibitory antibiotic exposure. Principal component analysis (PCA) was performed on z-score standardized log_2_ fold changes (−ΔΔCt) at 6 h. (**A**) PCA score plot showing biological replicates (*n* = 3 per condition) for rifampicin (RIF), rifampicin/minocycline (RIF/MIN) and dalbavancin (DAL) at 1/2× MIC or minocycline (MIN) at 1/4× MIC. (**B**) Loading plot shows the contribution of individual target genes to PC1 and PC2.

Exploratory analyses were performed to assess possible co-variation among selected regulator- and biofilm-associated transcripts (Figure A2). Increased *sarA* transcript abundance tended to coincide with increased *icaA*, *icaD*, and *fnbA* expression under rifampicin and minocycline exposure. No consistent pattern of co-variation between *icaR* and *icaA*/*icaD* expression was observed across conditions. This was particularly apparent under dalbavancin exposure, where lower *icaR* expression was not accompanied by corresponding changes in *icaA* or *icaD*. Furthermore, there was no consistent pattern of co-variation apparent between *vraS* and *sarA,* while scatterplots suggested a weak tendency toward co-variation between *vraS* and *lrgA* (Figure A2). Exploratory PCA indicated partial separation of the antibiotic-associated transcriptional profiles, with the corresponding score and loading plots shown in Figure A3.

Taken together, sub-inhibitory antibiotic exposure was associated with time-dependent, antibiotic-specific transcriptional profiles during early biofilm formation in *S. aureus* SA113. Rifampicin and minocycline were primarily associated with increased transcription of the selected adhesion- and PIA/PNAG-associated genes, whereas dalbavancin exposure was characterized by increased *vraS* and *lrgA* expression without relevant induction of *icaA*, *icaD*, or *fnbA*. Treatment-associated differences were generally less pronounced after 24 h than after 6 h.

## 4. Discussion

The aim of this study was to characterize the effects of sub-inhibitory antibiotic concentrations on bacterial adherence and early biofilm formation in the strong biofilm-forming *S. aureus* strain SA113, with particular emphasis on comparing responses to dalbavancin versus rifampicin and minocycline. A central observation of this study was that the antibiotics produced distinct drug-, concentration-, and time-dependent patterns of adherent biofilm biomass, surface colonization, and selected transcriptional responses, whereas culturable adherent bacterial counts remained broadly comparable across the tested sub-inhibitory antibiotic concentrations. The applied methods assess complementary aspects of the surface-associated phenotype. While crystal violet staining reflects the total retained adherent biomass, including bacterial and extracellular components, CFU enumeration quantifies culturable adherent bacteria and SEM provides a descriptive assessment of surface coverage and spatial organization. The discrepancy observed between stable CFU counts and altered adherent biofilm biomass aligns with prior findings that sub-inhibitory antibiotic exposure can modify biofilm accumulation and surface-associated organization without necessarily affecting population size [6,30]. Our observations are supported by the emerging concept that sub-inhibitory antibiotic concentrations can function as biologically active signals capable of modulating bacterial transcriptional and phenotypic responses rather than simply reflecting insufficient antimicrobial exposure [30]. Numerous studies have demonstrated that sub-MIC antibiotic exposure, including exposure to β-lactams, glycopeptides, and tetracyclines, can alter the expression of biofilm-associated genes in *S. aureus* in a strain-dependent manner [31,32,50]. Importantly, these regulatory effects may occur in the absence of pronounced growth inhibition and are associated with alterations in biofilm formation, stress adaptation, and the emergence of antibiotic resistance under prolonged exposure [30].

To investigate antibiotic-dependent phenotypic changes and transcriptional responses following sub-inhibitory exposure, *S. aureus* SA113 was selected as a well-established and frequently used strain with robust biofilm-forming capacity [10,23,24].

Rifampicin exposure showed increases in adherent biofilm and greater retention of surface-associated biomass, especially at higher sub-inhibitory concentrations, and was associated with comparatively dense surface colonization as observed by SEM. At 1/2× MIC, this phenotype coincided with significant *sarA* upregulation, increases in *icaA* and *icaD* transcript abundance, and increased *fnbA* expression (Figure 4). The capability of sub-inhibitory rifampicin to promote biofilm formation has previously been reported in several weak and non-producing *S. aureus* strains, where matrix composition analysis indicated a predominantly polysaccharide-based biofilm [33], which is biologically compatible with the increased *icaA* and *icaD* transcript abundance observed in our study; however, we did not directly quantify PIA/PNAG production.

Minocycline, in contrast, exerted variable concentration-dependent effects. Retained biomass markedly decreased at lower sub-inhibitory concentrations, but partially returned toward the control level at 1/2× MIC. This biphasic behavior may reflect the interplay between translational inhibition and concentration-dependent regulatory adaptation. Tetracycline-class inhibition of translation may restrict the synthesis of newly produced surface-associated proteins at lower sub-inhibitory concentrations [51,52], whereas stronger sub-inhibitory exposure may elicit regulatory responses that partially offset this effect, potentially increasing matrix-associated and overall adherent biofilm accumulation [31,53]. At 6 h, exposure at 1/4× MIC was associated with significant upregulation of *sarA*, *icaD*, and *fnbA*, alongside a moderate but non-significant increase in *icaA* expression; however, the mechanistic background of this behavior remains unresolved.

Rifampicin/minocycline co-exposure significantly reduced retained biomass at selected sub-inhibitory concentrations, including 1/8× MIC, but did not consistently reduce biomass further than minocycline mono-exposure at the corresponding relative concentrations. Rifampicin is commonly combined with other antibacterial agents because of the rapid emergence of resistance during monotherapy and the enhanced activity of selected combinations against staphylococcal biofilms and in implant-associated infection models [53,54,55]. However, these therapeutic objectives and experimental settings differ from the present model, which examined early surface accumulation during continuous sub-inhibitory exposure, and therefore need to be interpreted independently. Although rifampicin and tetracyclines have been reported to exhibit antibiofilm activity against established *S. aureus* biofilms at inhibitory concentrations [53,54,55], recent data indicate that their effects under sub-inhibitory conditions are not uniformly suppressive [33,56]. This distinction is important because activity against mature biofilms does not necessarily predict effects on initial attachment, early surface colonization, or biofilm-associated transcriptional responses at concentrations below the MIC. Likewise, conventional in vitro susceptibility does not necessarily predict therapeutic efficacy in biofilm-associated infections. In the present model, elevated *sarA* and *fnbA* transcript abundance during co-exposure was accompanied by only limited induction of *icaA* and *icaD*. Interestingly, this reduced induction of *icaA* and *icaD* was not associated with a correspondingly pronounced reduction in retained biomass compared with minocycline mono-exposure at 1/2× MIC. This non-uniform target-gene pattern highlights that the integrated 24-h phenotype assessed by CV staining cannot be directly inferred from the expression of individual genes at selected earlier time points, because biofilm-associated transcription is temporally dynamic, whereas CV staining provides an endpoint measure of retained surface-associated biomass without distinguishing between bacterial cells and extracellular material [56,57]. Accordingly, the transcriptional and phenotypic readouts should be interpreted as complementary rather than directly equivalent measures.

Dalbavancin exposure produced the clearest transcriptional contrast to rifampicin and minocycline. Previous work has primarily examined dalbavancin against established staphylococcal biofilms, prosthetic-joint-infection isolates, or in combination with other antibiotics, demonstrating activity against mature biofilm-associated bacteria under conditions that substantially differ from the early continuous sub-inhibitory exposure used here [38,41,42]. In the present model, sub-inhibitory exposure to dalbavancin increased *vraS* expression, consistent with a cell-wall-stress-associated transcriptional response. Activation of this two-component system by cell-wall-targeting antibiotics is well described and induces a cell-wall-stress stimulon involved in peptidoglycan synthesis and envelope remodeling [13]. In contrast, dalbavancin exposure increased *vraS* without a concomitant increase in *sarA* or induction of the selected matrix-associated genes *icaA* and *icaD*, while *fnbA* showed only a slight increase in transcript abundance. Thus, the dalbavancin-associated profile substantially differed from responses observed under rifampicin and minocycline exposure. Morphologically, this transcriptional profile coincided with pronounced bacterial clustering and reduced surface coverage (Figure 3), which may be associated with stress-adaptation responses in biofilms [13]. Taken together, the lack of *icaA*/*D* upregulation and increased expression of cell-wall-stress-associated genes were associated with reduced adherent biofilm accumulation under the tested conditions.

To summarize, our findings indicate that antibiotics highly effective against mature biofilms may not necessarily suppress early processes of adhesion and biofilm formation when present at sub-inhibitory concentrations. Furthermore, conventional MIC-based susceptibility reflects inhibition of planktonic bacterial growth under standardized conditions and should not be equated with therapeutic efficacy against biofilm-associated infection, where altered physiological states, matrix-associated tolerance, and pharmacokinetic factors may substantially affect treatment response. These findings align with previous reports showing that sub-inhibitory antibiotic concentrations can exert complex and sometimes paradoxical effects on biofilm formation. For example, sub-MIC rifampicin has been shown to increase biofilm formation in *S. epidermidis*, and similar effects have been described for other antibiotics, including erythromycin and tigecycline, often in association with increased *ica* expression and PIA/PNAG production [31,57,58]. Thus, such responses are not limited to a single antibiotic class but occur across agents with diverse mechanisms of action. While early studies in *S. aureus* suggested that sub-inhibitory exposure to antibiotics, including cell-wall-active agents, promotes biofilm formation [31,59,60], recent work indicates that these responses are highly heterogeneous and depend on strain background, antibiotic class and concentration, biofilm stage, and experimental conditions. This is further supported by observations in other bacterial species, such as *P. aeruginosa*, where very low antibiotic concentrations (~1/128× MIC) have been shown to suppress biofilm formation [61], highlighting that biofilm responses to sub-inhibitory exposure are not unidirectional.

From a translational perspective, our findings emphasize the importance of considering not only direct antibacterial activity, but also antibiotic-associated changes in bacterial adherence, biofilm accumulation and stress-associated transcriptional responses when designing antimicrobial biomaterials. Effective coatings should therefore achieve sufficiently high initial bactericidal concentrations while avoiding prolonged exposure to sub-inhibitory levels, for example, through release strategies combining strong burst release with sustained drug delivery at inhibitory concentrations.

Owing to its high biofilm-forming capacity, the present study used the strain *S.* aureus SA113 as a sensitive and reproducible model for detecting antibiotic-associated phenotypic and transcriptional responses [10,23,24]. Its *agr*-deficient background is associated with pronounced surface-associated accumulation by limiting quorum-sensing-mediated biofilm organization and dispersal [12,62], facilitating the detection of antibiotic-associated changes in retained adherent biomass under standardized conditions. However, broader generalizability requires validation in additional strains. Observed phenotypic alterations and transcriptional responses should be considered SA113-specific and cannot be assumed to represent responses of *agr*-competent or clinical *S. aureus* isolates without further validation. Future studies will therefore extend this approach to assess whether the observed response patterns are conserved across *agr*-competent strains and clinical isolates representing diverse genetic backgrounds. TSB, a nutrient-rich medium commonly used for *S. aureus* biofilm studies, provided a controlled environment for comparing antibiotic-associated effects while minimizing variability from host-derived factors [63]. However, it does not fully reproduce clinically relevant conditions, including host proteins, immune components, fluid flow, and nutrient or antibiotic gradients [64,65]. Accordingly, the phenotypic and transcriptional response patterns observed in this controlled model may differ under more physiologically complex conditions. Future studies should therefore incorporate components such as serum, plasma, fibrinogen, or fibronectin to improve physiological relevance. In addition, bacteria were continuously exposed to defined antibiotic concentrations, whereas local antimicrobial delivery systems typically generate dynamic profiles with an initial burst followed by declining concentrations [27,28,66]. The present experimental model therefore represents a controlled system to investigate the bacterial responses to prolonged sub-inhibitory antibiotic exposure. Accordingly, the present findings should be interpreted as responses to sustained, defined sub-inhibitory antibiotic exposure rather than as a representation of biomaterial-associated drug-release kinetics. Building on this model, future work will translate this approach to clinically relevant biomaterial-based systems with controlled antibiotic release, allowing direct assessment of how release kinetics modulate bacterial attachment, biofilm formation, and transcriptional adaptation. Finally, SEM and CV provide endpoint measures of biofilm morphology and accumulated adherent biomass. Direct correlations between qPCR data and CV or SEM findings should be interpreted cautiously, as qPCR measurements at 6 and 24 h represent discrete transcriptional states at the respective sampling times and concentrations, whereas CV staining integrates the cumulative accumulation of retained bacterial and extracellular material over the 24-h incubation period. SEM likewise provides a qualitative endpoint representation of surface organization. Accordingly, these datasets should be interpreted as complementary rather than as directly corresponding mechanistic readouts. Furthermore, the transcriptional analyses were based on three biological replicates and should therefore be considered exploratory, particularly given the number of target genes and treatment comparisons examined. Establishing causal relationships will require functional validation, including direct quantification of PIA/PNAG, extracellular DNA, and matrix-associated proteins, as well as targeted genetic approaches. Nevertheless, the observed expression patterns provide transcriptional insight into antibiotic-associated biofilm responses and serve as a basis for future targeted functional studies.

## 5. Conclusions

The present study provides transcriptional insight into cellular responses of the strongly biofilm-forming strain *S. aureus* SA113 to sub-inhibitory antibiotic exposure during early stages of biofilm formation. Together, the findings suggest that sub-inhibitory antibiotic exposure in SA113 does not uniformly promote or inhibit biofilm formation but rather is associated with antibiotic-specific patterns of adherent biofilm formation and transcriptional responses. Under the conditions examined, dalbavancin elicited a transcriptional response distinct from that observed with rifampicin and minocycline, characterized primarily by cell-wall stress-associated changes rather than induction of the selected matrix- and adhesion-related genes. This profile may be relevant for the development of dalbavancin-loaded biomaterials, although its potential functional advantage requires validation in release-based and biomaterial-associated biofilm models. To our knowledge, this study provides new insights into the early transcriptional responses of *S. aureus* to sub-inhibitory dalbavancin exposure during biofilm formation, as investigated in the model strain SA113. Validation in *agr*-competent strains and clinical isolates will be required to determine the broader generalizability of these response patterns.

## Figures and Tables

**Table 1:** Primer sequences and amplicon sizes used for qPCR.

Gene	Forward Primer (5′-3′)	Reverse Primer (5′-3′)	Amplicon Size (bp)
*gyrB*	TTGGTACAGGAATCGGTGGC	TCCATCCACATCGGCATCAG	86
*gmk*	GGACCATCTGGAGTAGGTAAAGG	TTCACCTTCACGCATTTGACG	111
*icaA*	ATGAACCGCTTGCCATGTGT	CTTCGTGTCCCCCTTGAGC	93
*icaD*	GAAACTATGGGCATTTTCGCGATT	CGATTCTCTTCCTCTCTGCCATTT	71
*icaR*	TTGCTGTTTCTTGAAAGTTGGT	AGTAGCGAATACACTTCATCTTGA	104
*fnbA*	AGCTTCCTTAGCTTCCGCTAC	ACACAAGTTGAAGTGGCACAG	90
*lrgA*	ATGCCAATTCCTATGCCTGC	CCGGCTGGTACGAAGAGTAA	143
*cidA*	TGCACCGTCTTCTACCCAAG	CCCTTAGCCGGCAGTATTGT	90
*atlA*	TGGCAGCTGATGTAGTTGGT	AACCGCACTGGTTGGGTAAA	73
*vraS*	ACCGGCAATATAACCTGCAC	CAACATGGAATTGGCGACCT	113
*sarA*	ACGTAATGAGCATGATGAAAGAACT	TGTTTGCTTCAGTGATTCGTTT	110
*sigB*	TGGGGCAACAAGATGACCATT	AACCGATACGCTCACCTGTC	150

Gene expression data are presented as log_2_ fold changes (−ΔΔCt) relative to the corresponding vehicle control after normalization to mean Ct value of the reference genes *gyrB* and *gmk*. Principal component analysis (PCA) was performed using z-score-standardized −ΔΔCt values.

## Data Availability

The data supporting the findings of this study are included in this published article and its Supplementary Information.

## Supplementary Materials

The following supporting information can be downloaded at: https://www.mdpi.com/article/10.3390/microorganisms14092101/s1.

## Abbreviations

The following abbreviations are used in this manuscript:

ATCC	American Type Culture Collection
CAMHB	Cation-adjusted Mueller–Hinton broth
CFU	Colony-forming unit
Ct	Cycle threshold
CV	Crystal violet
DAL	Dalbavancin
DMSO	Dimethyl sulfoxide
EPS	Extracellular polymeric substances
EUCAST	European Committee on Antimicrobial Susceptibility Testing
FnBPA/B	Fibronectin-binding proteins A/B
IcaR	Intercellular adhesion regulator
MIC	Minimum inhibitory concentration
MIN	Minocycline
MRSA	Methicillin-resistant *Staphylococcus aureus*
MRSE	Methicillin-resistant *Staphylococcus epidermidis*
OD600	Optical density at 600 nm
PBS	Phosphate-buffered saline
Ps-80	Polysorbate-80
PCA	Principal component analysis
PIA	Polysaccharide intercellular adhesin
PNAG	Poly-N-acetylglucosamine
qPCR	Quantitative polymerase chain reaction
RIF	Rifampicin
SarA	Staphylococcal accessory regulator A
SEM	Scanning electron microscopy
SD	Standard deviation
Tris	Tris(hydroxymethyl)aminomethane
TSB	Tryptic soy broth
VraSR	Vancomycin resistance-associated regulatory system
